# RBM15 activates glycolysis in M1-type macrophages to promote the progression of aortic aneurysm and dissection

**DOI:** 10.7150/ijms.97185

**Published:** 2024-07-22

**Authors:** Mi Tang, Min Wang, Zhirong Wang, Bo Jiang

**Affiliations:** Department of Cardiovascular Surgery, The Second Xiangya Hospital, Central South University, Changsha, Hunan Province, China.

**Keywords:** Aortic aneurysm and dissection, macrophages, glycolysis, RBM15, biomarkers

## Abstract

Aortic aneurysm and dissection (AD) represent a critical cardiovascular emergency with an alarmingly high mortality rate. Recent research has spotlighted the overexpression of genes associated with the m6A modification in AD patients, linking them to the presence of inflammatory M1-type macrophages. Moreover, glycolysis is widely recognized as a key feature of inflammatory M1-type macrophages, but biomarkers linking glycolysis and macrophage function to promote disease progression in AD have not been reported. We conducted an analysis of aortic immune cell infiltration, macrophages, and m6A-related biomarkers in AD patients using bioinformatics techniques. Subsequently, we employed a combination of RT-PCR, WB, and immunofluorescence assays to elucidate the alterations in the expression of M1- and M2-type macrophages, as well as markers of glycolysis, following the overexpression of key biomarkers. These findings were further validated *in vivo* through the creation of a rat model of AD with knockdown of the aforementioned key biomarkers. The findings revealed that the m6A-modified related gene RBM15 exhibited heightened expression in AD samples and was correlated with macrophage polarization. Upon overexpression of RBM15 in macrophages, there was an observed increase in the expression of M1-type macrophage markers CXCL9 and CXCL10, alongside a decrease in the expression of M2-type macrophage markers CCL13 and MRC1. Furthermore, there was an elevation in the expression of glycolytic enzymes GLUT1 and Hexokinase, as well as HIF1α, GAPDH, and PFKFB3 after RBM15 overexpression. Moreover, *in vivo* knockdown of RBM15 led to an amelioration of aortic aneurysm in the rat AD model. This knockdown also resulted in a reduction of the M1-type macrophage marker iNOS, while significantly increasing the expression of the M2-type macrophage marker CD206. In conclusion, our findings demonstrate that RBM15 upregulates glycolysis in macrophages, thus contributing to the progression of AD through the promotion of M1-type macrophage polarization. Conversely, downregulation of RBM15 suppresses M1-type macrophage polarization, thereby decelerating the advancement of AD. These results unveil potential novel targets for the treatment of AD.

## Introduction

Aortic aneurysm and dissection (AD) is a critical cardiovascular condition associated with a significantly high mortality rate 1-3. In general, the incidence rate is 3-5 cases per 100,000 people, with a high incidence rate of 10 cases per 100,000 in the middle-aged and elderly population 4, 5. Statistical studies have shown that if left untreated, it can kill as many as 50% of people within 48 hours 5. Common causes of AD include long-term uncontrolled hypertension, aortic coarctation, genetic disorders such as Marfan syndrome, and bileaflet aortic valve coarctation 6, 7. The pathogenesis of AD includes abnormal phenotypic transformation and apoptosis of vascular smooth muscle cells, abnormal degradation of extracellular matrix, endothelial dysfunction and immune cell infiltration 8-10. The m6A modification, characterized by the methylation of adenosine at the N6 position in RNA molecules, has emerged as a crucial epitranscriptomic regulator that influences various aspects of RNA metabolism, including splicing, stability, localization, and translation efficiency 11. Given the pivotal role of RNA regulation in cellular function and disease processes, dysregulation of m6A modification has been implicated in diverse pathological conditions, including cancer, neurodegenerative disorders, and inflammatory diseases 12, 13. In the last few years, an increasing number of studies have suggested that the m6A modification is involved in the progression of AD and is associated with inflammatory responses 14-16, but specific functional and mechanistic studies have not been reported.

Glycolysis, the metabolic process that converts glucose into pyruvate, is increasingly recognized for its pivotal role in regulating the inflammatory response, particularly in macrophages, which are key effector cells of the innate immune system 17, 18. In the context of inflammation, macrophages undergo metabolic reprogramming, shifting their energy metabolism towards glycolysis to meet the increased demand for energy and biosynthetic precursors required for their effector functions 19, 20. This metabolic switch not only provides macrophages with rapid energy production but also modulates their functional phenotypes 21-23. Moreover, one crucial regulator of glycolysis in macrophages is pyruvate kinase isoform M2 (PKM2) 24, 25. PKM2 plays a central role in coupling glycolytic flux with inflammatory responses 25. It has been demonstrated that PKM2 regulates the expression of pro-inflammatory cytokines, such as interleukin-1 beta (IL-1β), by modulating the activity of hypoxia-inducible factor 1 alpha (HIF1α), a key transcription factor involved in the inflammatory signaling pathway 26. Additionally, PKM2 activation promotes the activity of eukaryotic translation initiation factor 2 alpha kinase 2 (EIF2AK2), also known as PKR (protein kinase R), leading to enhanced inflammatory vesicle activity 27, 28. Dysregulation of PKM2-mediated glycolysis has been implicated in various inflammatory diseases, including cardiovascular diseases, highlighting its significance in the pathogenesis of human disorders 29.

Inducible nitric oxide synthase (iNOS) is another critical mediator linking metabolism and macrophage polarization 30, 31. iNOS catalyzes the production of nitric oxide (NO), which serves as a potent signaling molecule in the immune system. Importantly, NO generated by iNOS inhibits the polarization of M1-type macrophages to M2-type macrophages induced by interleukin-4 (IL-4) 32. This inhibition occurs through NO-mediated impairment of the mitochondrial electron transport chain in macrophages, disrupting the metabolic and functional transitions required for M2 polarization 33. This dysregulated macrophage polarization contributes to the perpetuation of inflammatory responses and the pathogenesis of various diseases characterized by dysregulated immune responses 34.

Drawing from the interconnectedness highlighted above, we have formulated a pivotal scientific inquiry: do genes associated with m6A modification exert influence on macrophage polarization, thereby propelling the progression of primary AD through glycolytic pathways? In pursuit of answers, our study harnesses bioinformatics methodologies to delve into this intricate relationship, meticulously quantifying the expression disparities of highly expressed genes implicated in m6A modifications within the context of AD, juxtaposed against both M1 and M2 macrophage phenotypes. Furthermore, we embark on experimental endeavors to dissect the multifaceted impacts of key genes implicated in m6A modification in AD, scrutinizing their effects on biomarker expression and glycolytic metabolism across M1 and M2 macrophage subtypes. Moreover, to bridge the gap between bench and bedside, we employ an SD rat model of AD, providing a dynamic *in vivo* platform to unravel the nuanced effects of m6A-modifying genes on AD progression, cognitive function, and macrophage polarization within the intricate milieu of the disease microenvironment. Through these integrated approaches, we aim to illuminate novel insights into the role of m6A modification in shaping the trajectory of AD, offering potential avenues for therapeutic intervention and clinical translation.

## Materials and methods

### Bioinformatics analysis

AD gene expression profiles were acquired from the Gene Expression Omnibus (GEO) database, with a specific focus on data generated using the Illumina HumanHT-12 V4.0 Expression Microbead Chip platform (accession number: GSE52093). Subsequent analysis encompassed the evaluation of immune cell infiltration, RBM15 expression in relation to macrophage subtypes, and the expression profile of m6A-related enzymes. These comprehensive analyses were executed utilizing RStudio (version 4.3.1) for both visualization and data processing, ensuring robust and accurate interpretation of the acquired data.

### RT-PCR

Total RNA extraction from vascular tissues obtained from both the AD and normal groups was carried out using TRIzol reagent (Lot: #9108, Takara, Japan), following the manufacturer's protocol. Subsequently, cDNA synthesis was performed via reverse transcription, employing the Fast One Step Probe RT-PCR Kit (Lot: # RK20413, ABclonal, China). This method facilitated simultaneous PCR amplification of the target sequences. The primer sequences employed in the PCR reactions were meticulously documented and are provided in **Supplementary Table 1** for reference.

### Tissue immunofluorescence

Vascular tissues obtained from both AD and normal groups underwent fixation in 4% paraformaldehyde for a duration of 24 hours, followed by dehydration, routine embedding, and sectioning. Post-fixation, antigen retrieval procedures were carried out, and subsequently, the sections were incubated for 30 minutes for proper closure. Primary antibodies, including CD206 antibody (Lot: YA798, MCE, USA) and iNOS antibody (Lot: HY-P80725, MCE, USA), were applied, followed by incubation with secondary antibodies, specifically goat anti-rabbit IgG (H+L) highly cross-adsorbed secondary antibody conjugated with Alexa Fluor™ 488 (Lot: A-11034, Thermo Fisher, USA). Incubation of the antibodies took place in an oven set at 37°C for 30 minutes. Following antibody incubation, DAPI (Lot: D1306, Thermo Fisher, USA) was utilized for restaining the samples, with an additional incubation period of 10 minutes at room temperature. The sections were then subjected to blocking procedures before microscopic examination (OLYMPUS, BX51) and subsequent photography.

### Cell culture

THP-1 cells, procured from the Shanghai Cell Bank, Chinese Academy of Sciences, were utilized in this study. To induce maturation towards macrophages, Phorbol 12-myristate 13-acetate (PMA) (Lot: 16561-29-8, absin, China) was added to complete medium comprising RPMI-1640 (Gibco, Cleveland, TN, USA), supplemented with 10% fetal bovine serum (FBS; Gibco) and 1% penicillin-streptomycin (P/S). This induction process facilitated the differentiation of THP-1 cells into macrophages, a crucial step in subsequent experimental procedures.

### Construction and transfection of sh-RBM15 and Lv-RBM15 plasmids

Lv-RBM15 and negative control sequences were incorporated into the overexpression vector GV135 (Giga Bio, Shanghai, China), while sh-RBM15 and negative control shRNA sequences were integrated into the adenoviral vector pUCM-AAVS1-TO-hNGN2 (Giga Bio, Shanghai, China). THP-1 cells were transfected with Lv-RBM15 (abm, Jiangsu, China) following the manufacturer's protocol, and subsequent screening was conducted using puromycin (Lot: ST551-10 mg, Biyun Tian, China) to establish stable transfections. To achieve knockdown of RBM15 in AD model mice, adenovirus-infected sh-RBM15 and negative control shRNA were administered. Detailed sequences for Lv-RBM15, sh-RBM15, and their respective controls can be found in **Supplementary Table 2** for reference.

### Western blotting

THP-1 macrophages and macrophages overexpressing RBM15 were cultured in 6-well plates until reaching a confluency of 85% or higher. Total proteins were then extracted using lysis buffer containing radioimmunoprecipitation assay (RIPA), and subsequent experimental procedures were conducted following standard Western blot methods. For Western blot analysis, the following antibodies were utilized: hexokinase (Lot: #2804, CST, USA), GAPDH (Lot: AC002, ABclid, China), anti-mouse recombinant secondary antibody (Lot: RGAM001, proteintech, China), and anti-rabbit recombinant secondary antibody (Lot: RGAR001, proteintech, China). These antibodies were employed to detect specific target proteins and facilitate visualization of protein expression levels.

### ELISA

The THP-1 macrophage cell line was grown to 80% density, washed three times with 1x PBS (pH 7.4), and then added to 1 mL of complete medium for immune challenge stimulation. Macrophages were stimulated with 20 ng/mL lectin LPS for 24 hours to produce IL6 and TNF. The cell supernatants from the stimulation were collected and stored at -80°C. Rabbit antibodies specific for IL6 and TNF were added to plates and incubated overnight at 4°C. After removing unbound antibodies, blank wells were treated with BSA enzyme inhibitor, and macrophage supernatant was added. The plate was incubated at room temperature, washed to remove excess proteins, and then specific secondary antibodies and substrates were added. The reaction was stopped using a stop solution, and absorbance values were read to determine the levels of IL6 and TNF in the samples.

### Establishment of an AD model in SD rats

SD rats aged four weeks were subjected to an experimental protocol aimed at inducing aneurysm formation. β-aminopropionitrile (BAPN), obtained from MCE, USA (Lot: A3134), was administered in the drinking water at a dose of 1 g/kg. To ensure sustained exposure, the drinking water was refreshed twice a week, with the BAPN concentration incremented by 0.025% per change, over a duration of four weeks. Following this pre-treatment phase, Angiotensin II (Ang II) sourced from MCE, USA (Lot: 4474-91-3), was injected intraperitoneally at a dosage of 4 mg/kg, administered once in the morning and once in the evening 35. The rats were carefully monitored throughout the experiment for any signs of distress or adverse effects. Upon reaching the experimental endpoint or humane criteria, rats were euthanized according to approved protocols, and their aortic arches were meticulously excised for subsequent pathological examination. Ethical considerations were strictly adhered to, in accordance with the guidelines established by the Animal Welfare Committee and The Second Xiangya Hospital, Central South University Ethical Committee (Approval number: SQ2025746). This experimental design aims to elucidate the mechanisms underlying aneurysm development and evaluate potential therapeutic interventions in a manner that ensures the welfare and ethical treatment of the animals involved.

### Flow cytometry

Arteries obtained from AD model mice were carefully excised and subjected to digestion with trypsin (Lot: R001100, Thermo Fisher, USA) to generate single-cell suspensions. The resulting cells were then washed twice with phosphate-buffered saline (PBS) and subsequently incubated with 1% bovine serum albumin (BSA) buffer (Lot: 9048-46-8, Aladdin, China) for 30 minutes to block non-specific binding sites. Following the blocking step, the cells were further incubated with specific antibodies targeting iNOS (Lot: 17-5920-82, Thermo Fisher, USA) and CD206 (Lot: 17-2061-82, Thermo Fisher, USA) in 1% BSA buffer for an additional 30 minutes. This facilitated the identification and characterization of macrophage M1 and M2 phenotypes through flow cytometry analysis, enabling the determination of changes in macrophage polarization states within the arterial tissues.

### Statistical analyses

For the bioinformatics analyses, statistical assessments were conducted using the R language (version 3.2.1). Each statistical test was executed as a two-tailed test, with p-values less than 0.05 considered statistically significant. This rigorous approach ensured robust evaluation and interpretation of the bioinformatics data. In the experimental section, data were presented as mean ± standard deviation derived from three independent replicates. Statistical analyses were carried out using GraphPad Prism 8.0 software. Student's t-test or one-way analysis of variance (ANOVA) was employed for comparisons between groups, with p-values less than 0.05 indicative of statistical significance. This meticulous statistical analysis enabled accurate assessment of experimental results and identification of significant findings.

## Results

### RBM15 is highly expressed in AD and associated with macrophage polarization

**Figure 1:**
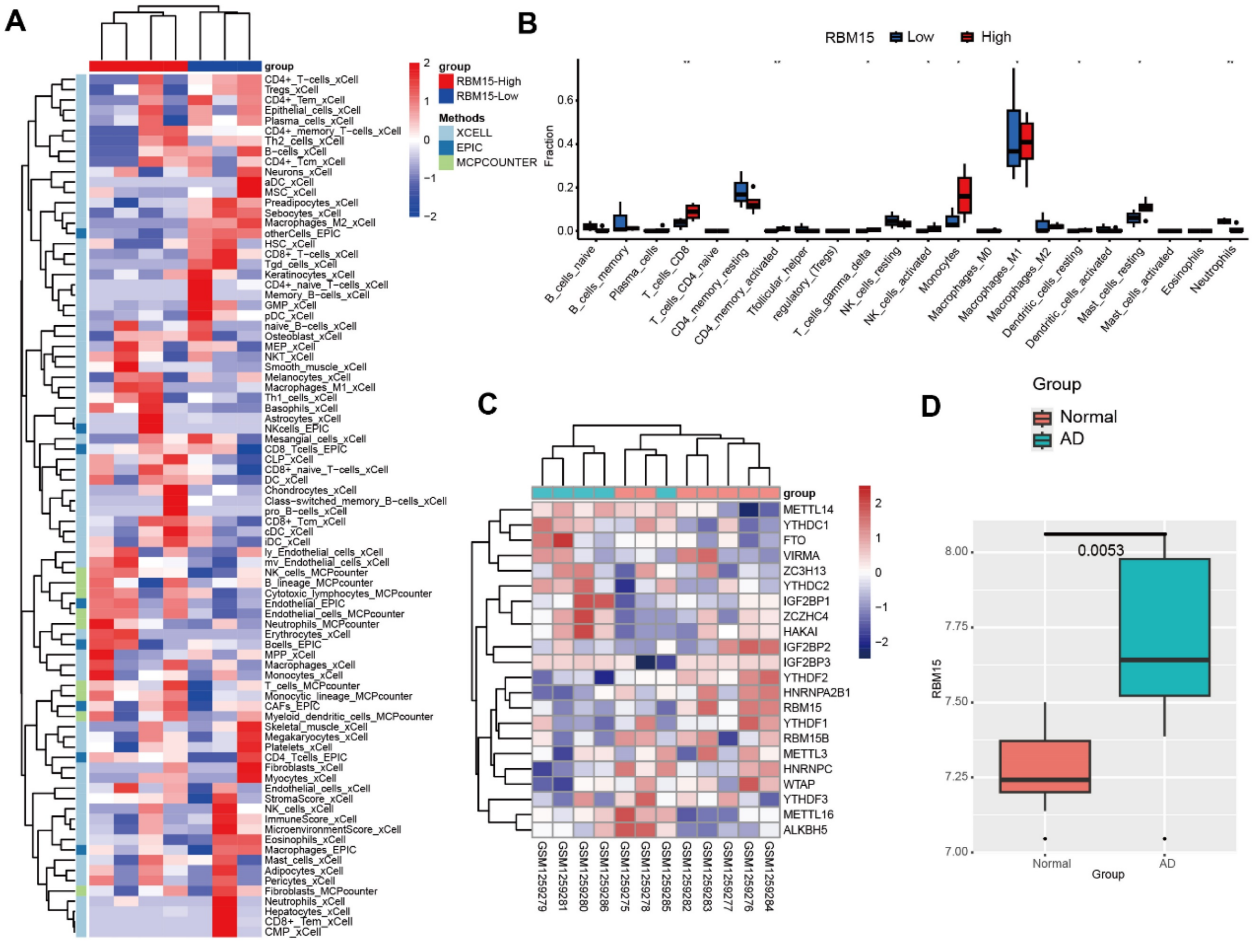
Bioinformatic analysis of AD gene expression profiles. (A) Heatmap of aortic immune cell infiltration, red: RBM15 high, blue: RBM15-low. (B) Histogram of aortic immune cell infiltration, red: RBM15-high, blue: RBM15-low. (C) Heatmap of expression of m6A-associated enzyme profiles in normal and AD groups. (D) Box plots of RBM15 expression in AD and normal groups. (* p < 0.05; ** p < 0.01).

**Figure 2:**
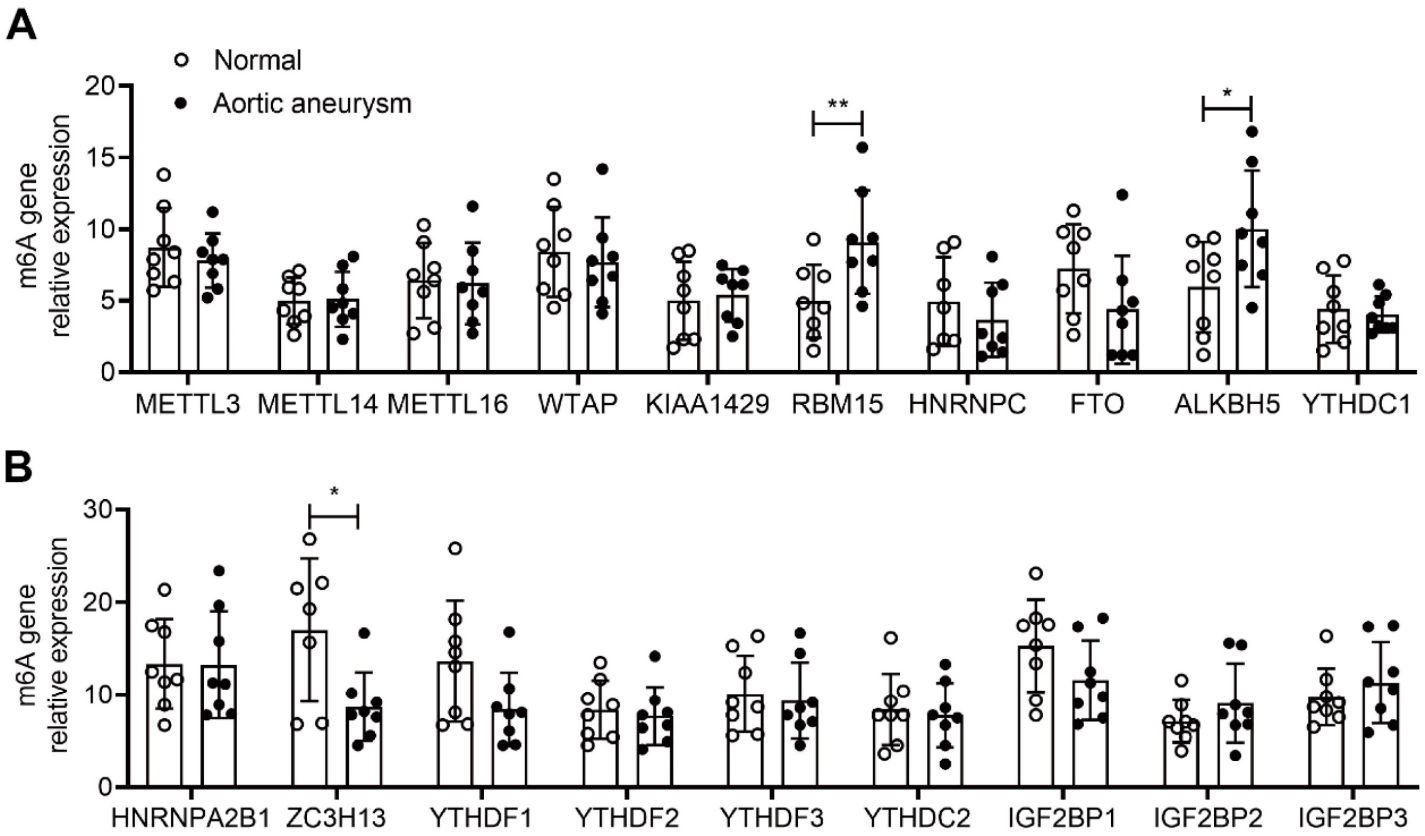
RT-PCR detection of mRNA expression of m6A-associated enzyme profiles. (A-B) mRNA expression of different m6A-associated enzyme profiles in AD and normal groups. (* p < 0.05; ** p < 0.01).

To investigate the expression and significance of RBM15 in aortic coarctation, we retrieved the microarray dataset GSE52093 from the GEO database, comprising gene expression profiles of aortic coarctation (n=7) and controls (n=5), and conducted bioinformatic analysis. Following analysis, we generated a heatmap illustrating aortic immune cell infiltration. Notably, the RBM15 high expression group exhibited significantly elevated levels of macrophage M1 subtype infiltration, accompanied by a marked reduction in M2 subtype infiltration **(Figure 1A)**. Moreover, our findings revealed a pronounced increase in macrophage M1 subtype infiltration in the RBM15 high expression group **(Figure 1B)**. Additionally, analysis of the m6A-associated enzyme profiles showcased a substantial upregulation of RBM15 expression in both the normal and AD groups **(Figure 1C)**. Furthermore, our study uncovered a significant elevation of RBM15 expression in AD compared to normal tissues **(Figure 1D)**. Subsequent verification via RT-PCR demonstrated significantly higher expression levels of RBM15 and ALKBH5 in vascular tissues from AD patients compared to those from the normal group, with RBM15 exhibiting the most pronounced increase** (Figure 2A-2B)**.

### M1 macrophages more polarized than M2 in AD rats

**Figure 3:**
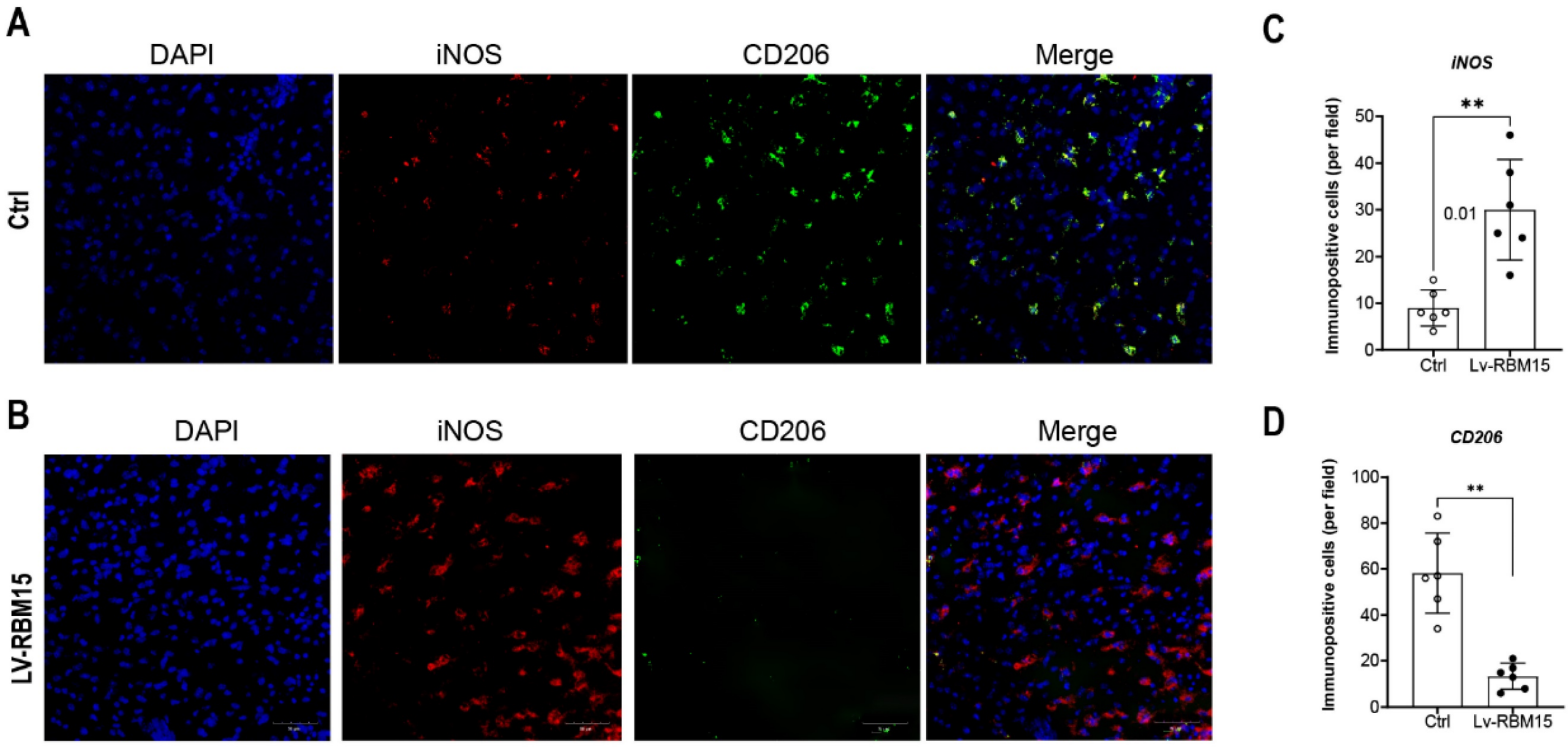
Immunofluorescence staining of vascular tissue. (A) Immunofluorescence staining graph of M1 subtype of vascular tissues in AD and normal groups, blue (DAPI, nucleus) and green (iNOS), (B) Staining statistics graph. (C) Immunofluorescence staining graph of vascular tissue M2 subtype in AD and normal groups, red (CD206), (D) M2 subtype staining statistic graph. (* p < 0.05; ** p < 0.01).

**Figure 4:**
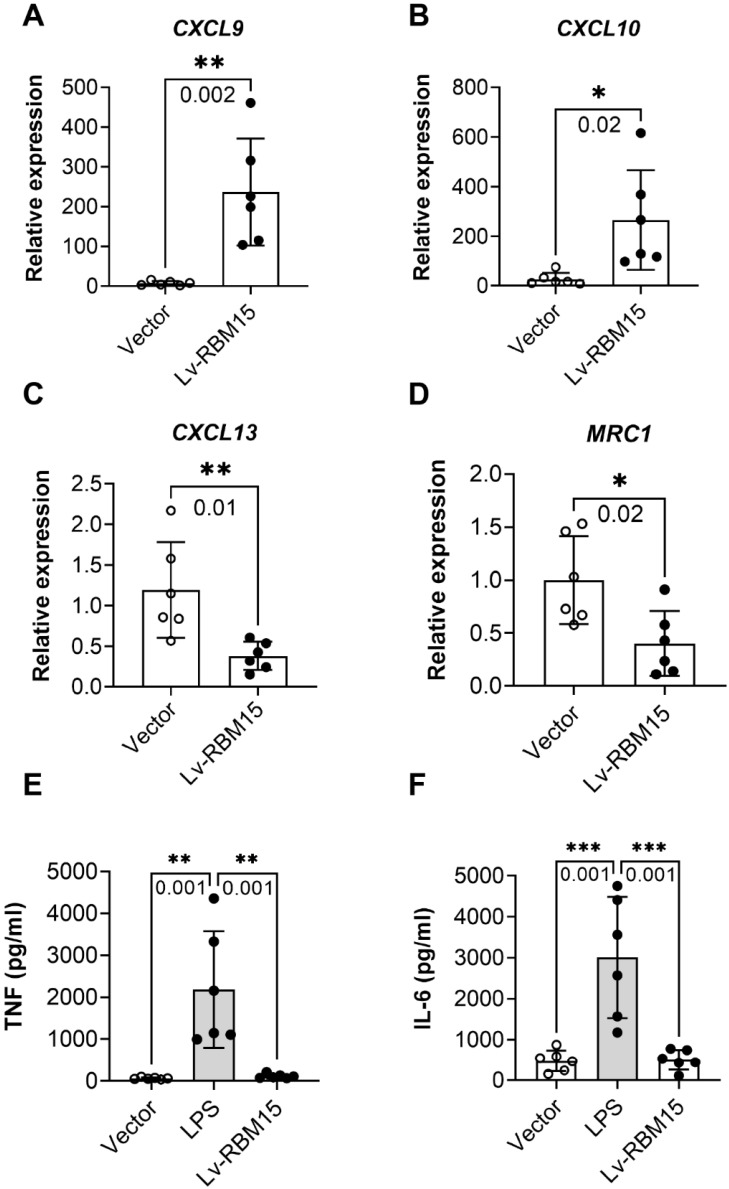
RT-PCR to detect the expression of macrophage M1- and M2-type related markers after overexpression of RBM15. Macrophage M1-related markers: (A) CXCL9 and (B) CXCL10; macrophage M2-related markers: (C) CXCL13 and (D) MRC1; other macrophage-related markers: (C) TNF and (D) IL-6. (* p < 0.05; ** p < 0.01, *** p < 0.001).

To investigate the impact of macrophage polarization on both AD rat and the normal group, we conducted immunofluorescence staining of vascular tissues from rats in these two groups. Our findings revealed a significant increase in the fluorescent expression of the M1 subtype (iNOS+) in both the AD and normal groups **(Figure 3A)**, while the fluorescent expression of the M2 subtype (CD206+) exhibited a marked decrease** (Figure 3C)**. Subsequently, quantification of the fluorescent expression of iNOS+ and CD206+ using image analysis software confirmed statistically significant differences, with increased iNOS+ **(Figure 3B)** and decreased CD206+ **(Figure 3D)** levels observed. Furthermore, in this study, we cultured macrophages overexpressing RBM15 using lentiviral transfection techniques, alongside normal macrophages, *in vitro*. Subsequent RT-PCR analysis revealed a significant upregulation in the expression of M1-associated markers CXCL9 **(Figure 4A)** and CXCL10 **(Figure 4B)** following RBM15 overexpression. Conversely, the expression levels of M2-associated markers CXCL13 **(Figure 4C)** and MRC1 (Figure 4D) were significantly decreased. Notably, RBM15 overexpression did not affect the expression of TNF **(Figure 4E)** and IL-6** (Figure 4F)**.

### RBM15 increases glycolysis in macrophages

**Figure 5:**
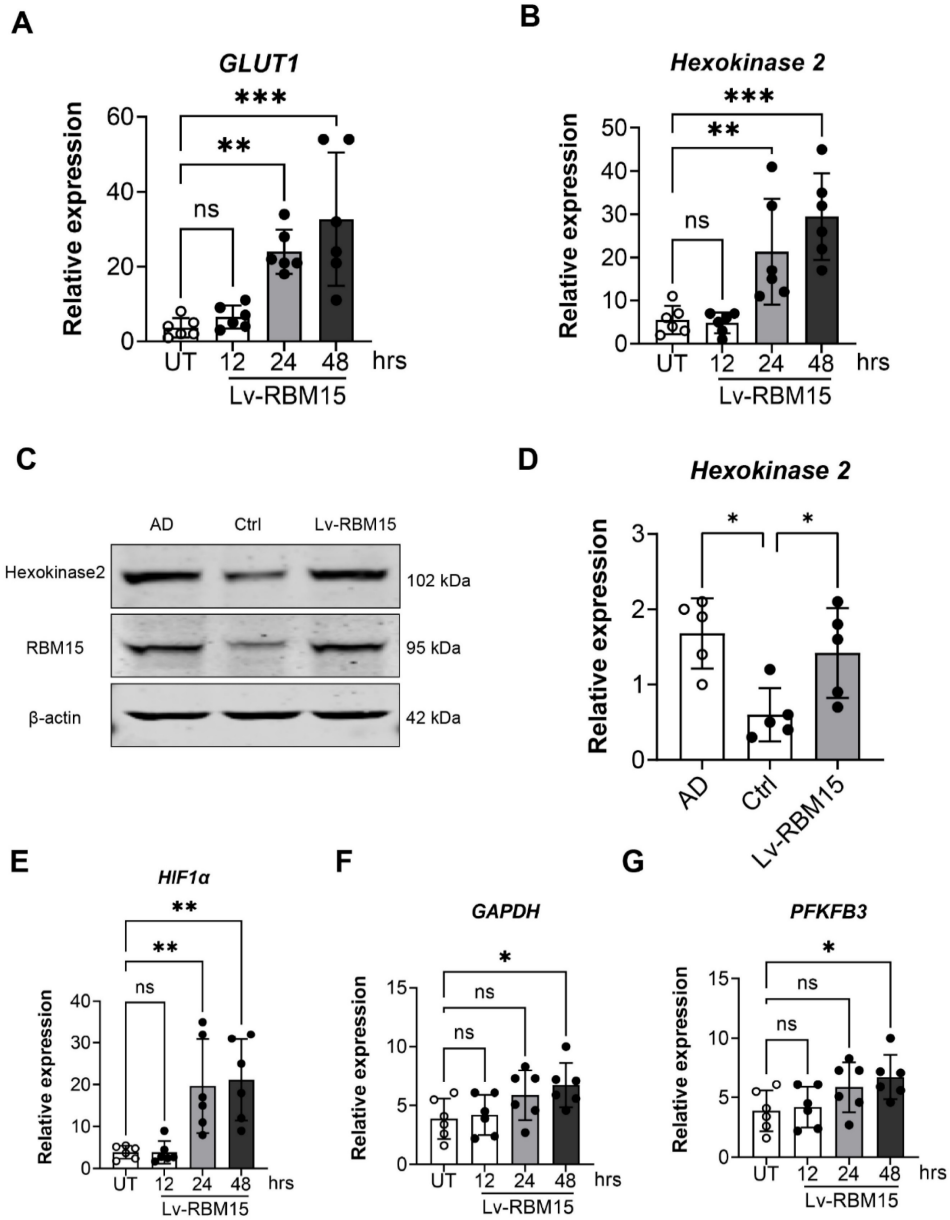
WB and RT-PCR to detect the expression of glycolysis key enzyme genes after overexpression of RBM15. RT-PCR to detect the expression of glycolysis key enzyme genes (A) GLUT1, (B) hexokinase mRNA after overexpression of RBM15; WB and RT-PCR to detect the expression of (C) hexokinase protein after overexpression of RBM15, (D) hexokinase 2 mRNA expression. RT-PCR to detect the expression of glycolysis key enzyme genes (E) HIF1α, (F) GAPDH and (G) PFKFB3 mRNA after overexpression of RBM15. (* p < 0.05; ** p < 0.01, *** p < 0.001).

To delve deeper into the influence of RBM15 on macrophages, we utilized lentiviral transfection techniques to overexpress RBM15, subsequently assessing the expression of key glycolysis enzymes, GLUT1 and hexokinase, via RT-PCR analysis. Our findings revealed a significant upregulation in the expression levels of the glycolytic enzymes GLUT1 **(Figure 5A)** and hexokinase **(Figure 5B)** following RBM15 overexpression. Moreover, we investigated hexokinase protein expression through Western blot analysis, which demonstrated a notable increase in hexokinase protein levels post-RBM15 overexpression, with the difference being statistically significant **(Figure 5C-5D)**. Furthermore, utilizing RT-PCR, we evaluated the expression of additional glycolysis-related enzymes. The results unveiled a significant upregulation in the expression levels of HIF1α **(Figure 5E)**, GAPDH** (Figure 5F)**, and PFKFB3 **(Figure 5G)** subsequent to RBM15 overexpression.

### RBM15 regulates macrophage polarization via the glycolytic pathway

**Figure 6:**
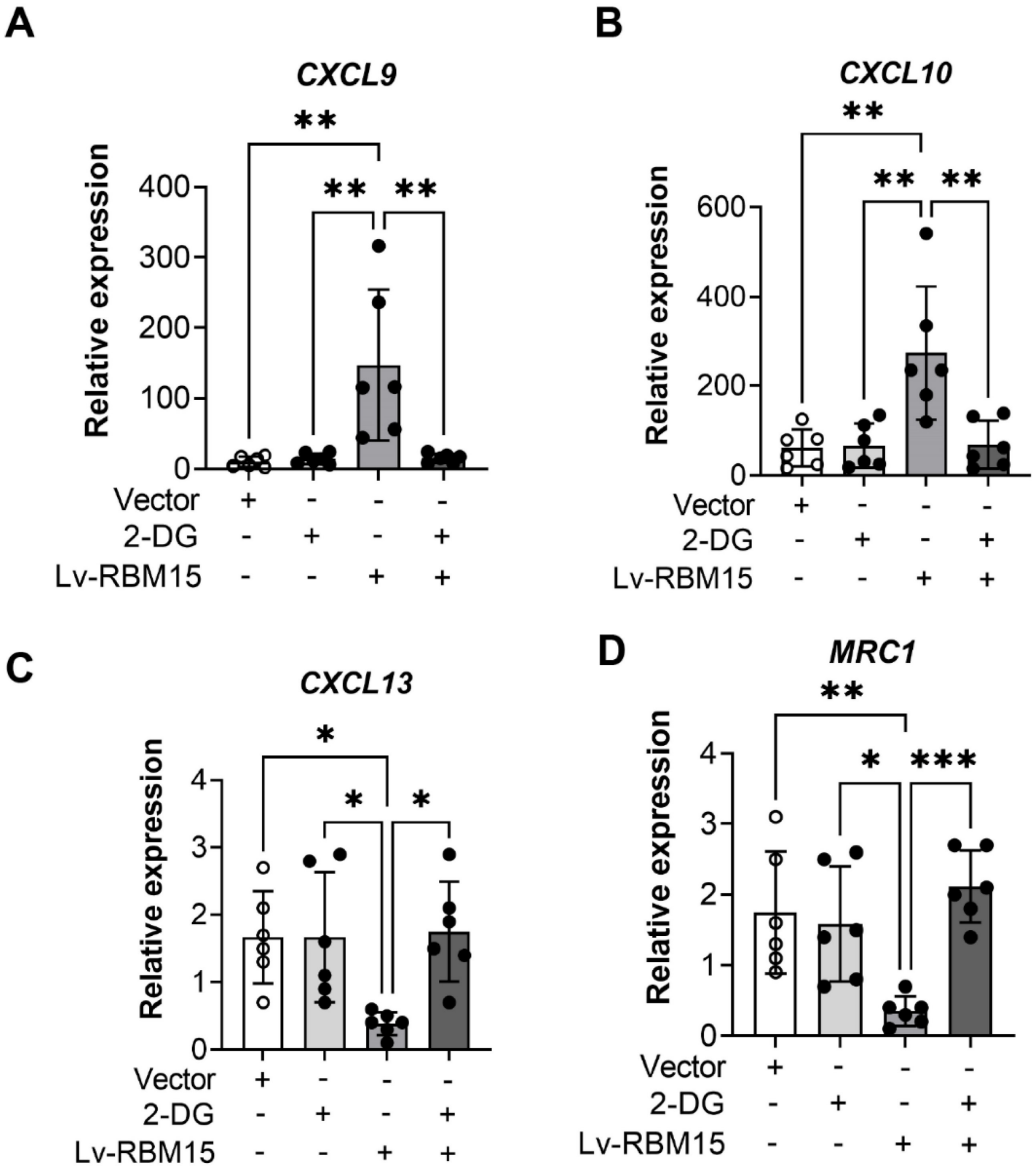
RT-PCR was performed to detect the effect of overexpression of RBM15 on the glycolytic pathway. Expression of M1-type macrophage-associated markers (A) CXCL9 and (B) CXCL10 after overexpression of RBM15 and addition of 2-DG; expression of M2-type macrophage-associated markers (C) CXCL13 and (D) MRC1 after overexpression of RBM15 and addition of 2-DG. (* p < 0.05; ** p < 0.01, *** p < 0.001).

To investigate whether RBM15 modulates macrophage polarization via the glycolytic pathway, we employed lentiviral transfection to overexpress RBM15 while concurrently supplementing the culture medium with the glycolytic inhibitor 2-DG for validation purposes. Our findings revealed that RBM15 overexpression led to an upregulation in the expression of M1 subtype-related markers CXCL9 and CXCL10, whereas the addition of 2-DG reversed this expression pattern** (Figure 6A-6B)**. Similarly, RBM15 overexpression resulted in decreased expression levels of the M2 subtype-related markers CXCL13 and MRC1, which were also restored upon treatment with 2-DG **(Figure 6C-6D)**.

### RBM15 knockdown improves aortic aneurysms in a rat model of AD

**Figure 7:**
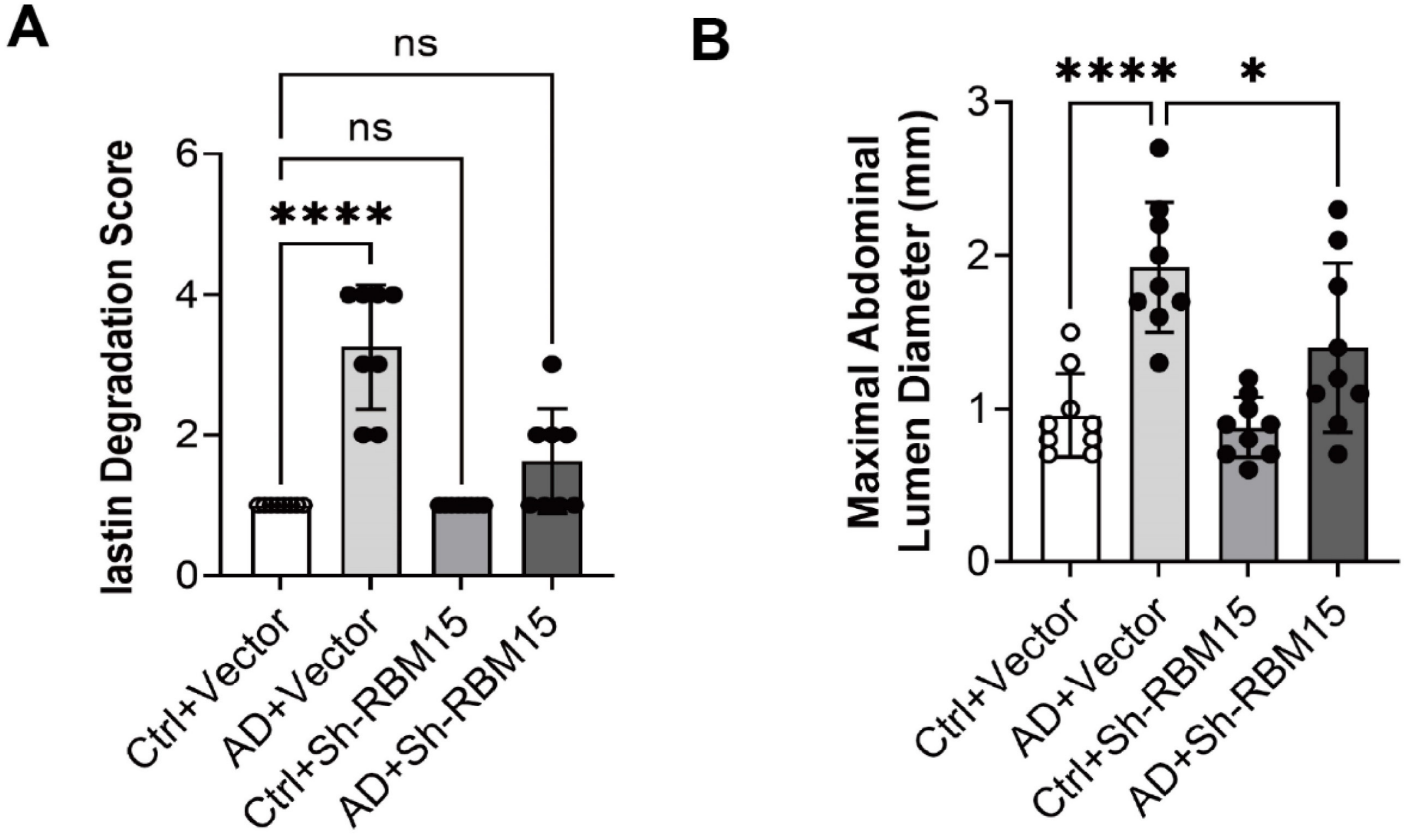
Effects of RBM15 knockdown on aortic aneurysms in the SD rat model of AD. (A) HE staining was used to detect the severity of aortic aneurysms; (C) elastin degradation score was used to evaluate the severity of aortic aneurysms; (B) gross photographs of the abdominal aorta in the four groups; and (D) determination of the diameter of the abdominal aorta indicated the severity of aortic aneurysms. (* p < 0.05; **** p < 0.0001).

We successfully established an AD model in SD rats and knocked down RBM15 using adeno-associated virus transfection to further validate the effect of RBM15 on AD progression *in vivo*. Firstly, we collected the arteries from the AD model rats for HE staining and then evaluated the severity of aortic aneurysm by elastin degradation score, the results showed that the aortic aneurysm in the AD+vector group was more severe than that in the Ctrl+vector group, indicating that the modelling was successful, and that the AD+sh-RBM15 group had a significant reduction in aortic aneurysm generation compared with the AD+vector group. Aortic aneurysm formation was significantly reduced in the AD+sh-RBM15 group **(Figure 7A, 7C)**. In addition, we collected gross photographs of abdominal aortic aneurysms from four groups **(Figure 7B)**, and by determining abdominal aortic aneurysm diameters, we found that abdominal aortic aneurysms were significantly more severe in the AD+Vector group than in the Ctrl+Vector group, indicating successful modelling, while abdominal aortic aneurysm diameters were significantly reduced in the AD+sh-RBM15 group compared with the AD+Vector group **(Figure 7D)**.

### RBM15 regulates macrophage polarization *in vivo*

**Figure 8:**
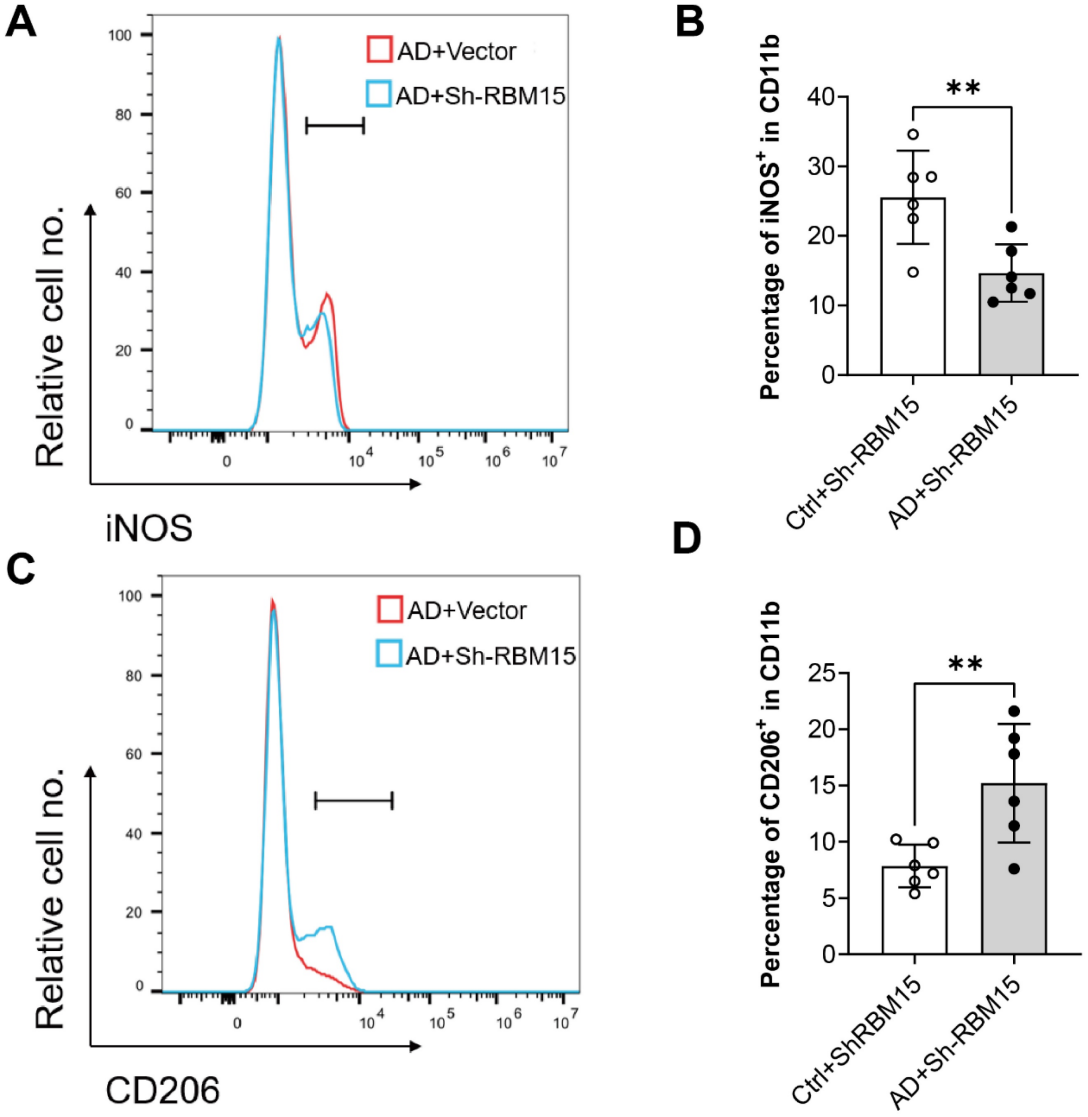
Expression of M1 and M2 type-related markers in arterial macrophages after knockdown of RBM15 in the SD rat model of AD was examined by flow cytometry. (A) Expression of the M1 macrophage marker iNOS and (B) fluorescence intensity analysis of iNOS expression, (C) expression of the M1 macrophage marker CD206 and (D) fluorescence intensity analysis of CD206 expression. (** p < 0.01).

To evaluate the impact of RBM15 on macrophages *in vivo*, we conducted flow cytometric analysis using an established AD model in SD rats, wherein RBM15 knockdown was performed. Subsequently, we assessed the polarization state towards the M1 phenotype. Our findings revealed a significant reduction in the M1-type macrophage marker iNOS expression in the aorta of the Ctrl+sh-RBM15 group compared to the AD+sh-RBM15 group following RBM15 knockdown** (Figure 8A-8B)**. Conversely, the expression of the M2-type macrophage marker CD206 in the aorta exhibited a significant increase in the Ctrl+sh-RBM15 group relative to the AD+sh-RBM15 group** (Figure 8C-8D)**.

## Discussion

Aortic dissection (AD) is a critical cardiovascular emergency, with growing evidence highlighting the crucial role of macrophage infiltration in the aortic wall, significantly affecting disease progression 36, 37. Despite this understanding, the precise mechanisms driving AD progression and associated biomarker changes remain unclear and underexplored 38. It is essential to investigate whether specific key biomarkers can either promote or hinder disease advancement by modulating macrophage infiltration through complex interactions. In this context, our study examined the intricate mechanisms at play, identifying the m6A-related gene RBM15 as significantly upregulated in AD patients and correlated with macrophage polarization through comprehensive bioinformatics analyses. Further investigations revealed that both overexpression and knockdown of RBM15 significantly influenced the expression profiles of macrophage polarization markers, including CXCL9, CXCL10, CXCL13, and MRC1. Additionally, RBM15 was found to intricately affect macrophage metabolic reprogramming, particularly by modulating glycolysis pathways. This discovery highlights the metabolic regulation within macrophages, presenting potential therapeutic avenues for AD intervention.

In recent years, there has been a growing interest in understanding the impact of m6A-related enzyme gene expression on the progression of AD. Research has demonstrated a significant difference in the percentage of m6A-modified mRNAs between AD tissue samples and healthy aortas, indicating a distinct molecular landscape in the pathology of the disease. Specifically, studies suggest that METTL14-mediated mRNA methylation and FTO-mediated mRNA demethylation work together to contribute to AD progression through complex molecular interactions. This highlights the intricate regulatory mechanisms orchestrated by m6A-related enzymes in the context of AD, providing valuable insights into potential therapeutic targets and intervention strategies 39. Our study began with a thorough bioinformatics analysis, revealing a significant upregulation of the m6A-related enzyme gene RBM15 in AD samples. Intriguingly, RBM15 expression was found to be elevated in M1 subtypes of macrophages, while showing a concurrent decrease in M2 subtypes. This observation sheds light on the intricate interplay between RBM15 expression and macrophage polarization states, highlighting its potential involvement in the pathogenesis of AD. Furthermore, RBM15 has been identified as an RNA-binding protein that affects cell growth by regulating signals that promote apoptosis, particularly in the hematopoietic Notch and Wnt pathways 40. In the field of cancer research, RBM15 has also been shown to impact the progression of various cancers, including squamous cell carcinoma 41, osteosarcoma 42, ovarian cancer 43 and lung cancer 44, by interacting with different proteins. Collectively, our study and previous research indicate that RBM15 plays a pivotal role in a range of diseases and may have influenced the progression of AD by affecting macrophage polarization.

The role of macrophages in promoting inflammation in the aortic wall has been extensively documented. Specifically, M1-type macrophages are associated with pro-inflammatory reactions, while M2-type macrophages are known for their anti-inflammatory properties and involvement in tissue repair. This dichotomy emphasizes the complex and dynamic nature of macrophage polarization in regulating the inflammatory environment within the aortic wall 45, 46. Studies in mouse models of AD have confirmed the infiltration of macrophages into the aorta, with macrophages emerging as the predominant cell type. However, the exact mechanisms governing this phenomenon and the associated changes in markers remain unclear, necessitating further investigation. Understanding these intricate mechanisms holds promise for advancing our comprehension of AD pathophysiology and potential therapeutic strategies 47. In our study, we observed increased iNOS expression and decreased CD206 expression in the vascular tissues of AD model mice, indicating an abundance of M1-type macrophages in these tissues. Furthermore, overexpression of RBM15 in the THP-1 macrophage cell line led to an upregulation of M1-type macrophage-associated markers CXCL9 and CXCL10, accompanied by a downregulation of M2-type macrophage-associated markers CXCL13 and MRC1. These findings suggest that m6A modifications may influence macrophage polarization, promoting a shift towards the M1-type state and potentially exacerbating AD progression through enhanced pro-inflammatory M1-type macrophage responses. However, further exploration is warranted to comprehensively elucidate the underlying mechanisms.

Glycolysis plays a pivotal role in the metabolic pathway of macrophages. The pro-inflammatory M1 macrophages primarily rely on glycolysis, while suppressing the TCA cycle and mitochondrial oxidative phosphorylation. Conversely, the anti-inflammatory M2 macrophages favor mitochondrial oxidative phosphorylation. Gerloff et al. demonstrated that fatty acid oxidation significantly influences the polarization of anti-inflammatory M2 macrophages, whereas inflammatory M1 macrophages heavily rely on glycolysis 48. This distinction emphasizes the metabolic adaptations associated with macrophage polarization and their unique functions in immune regulation and tissue homeostasis 27, 49. In our investigation, we observed an elevation in the expression of the glycolytic enzymes GLUT1 and hexokinase following RBM15 overexpression. Furthermore, regulatory elements such as HIF1α, GAPDH, and PFKFB3 were also upregulated. These observations suggest that increased levels of GLUT1 and hexokinase enhance cellular glucose uptake and glycolysis, supporting the energy requirements and activity of macrophages. Additionally, the upregulation of HIF1α, GAPDH, and PFKFB3 likely further stimulates the glycolytic pathway, promoting metabolic reprogramming and macrophage function. Furthermore, the use of 2-deoxy-D-glucose (2DG) inhibits glucokinase activity, ultimately disrupting macrophage glycolysis. This inhibition underscores the importance of glycolysis in macrophage function and underscores the potential therapeutic implications of targeting this metabolic pathway to modulate immune responses 50. Moreover, our study revealed that the alterations in the expression of M1-type (CXCL9 and CXCL10) and M2-type (CXCL13 and MRC1) macrophage markers induced by RBM15 overexpression could be reversed by 2DG treatment. These findings strongly support the concept that RBM15 regulates macrophage polarization through glycolysis, and the potential correlation of this process with AD progression warrants further investigation.

**Figure 9:**
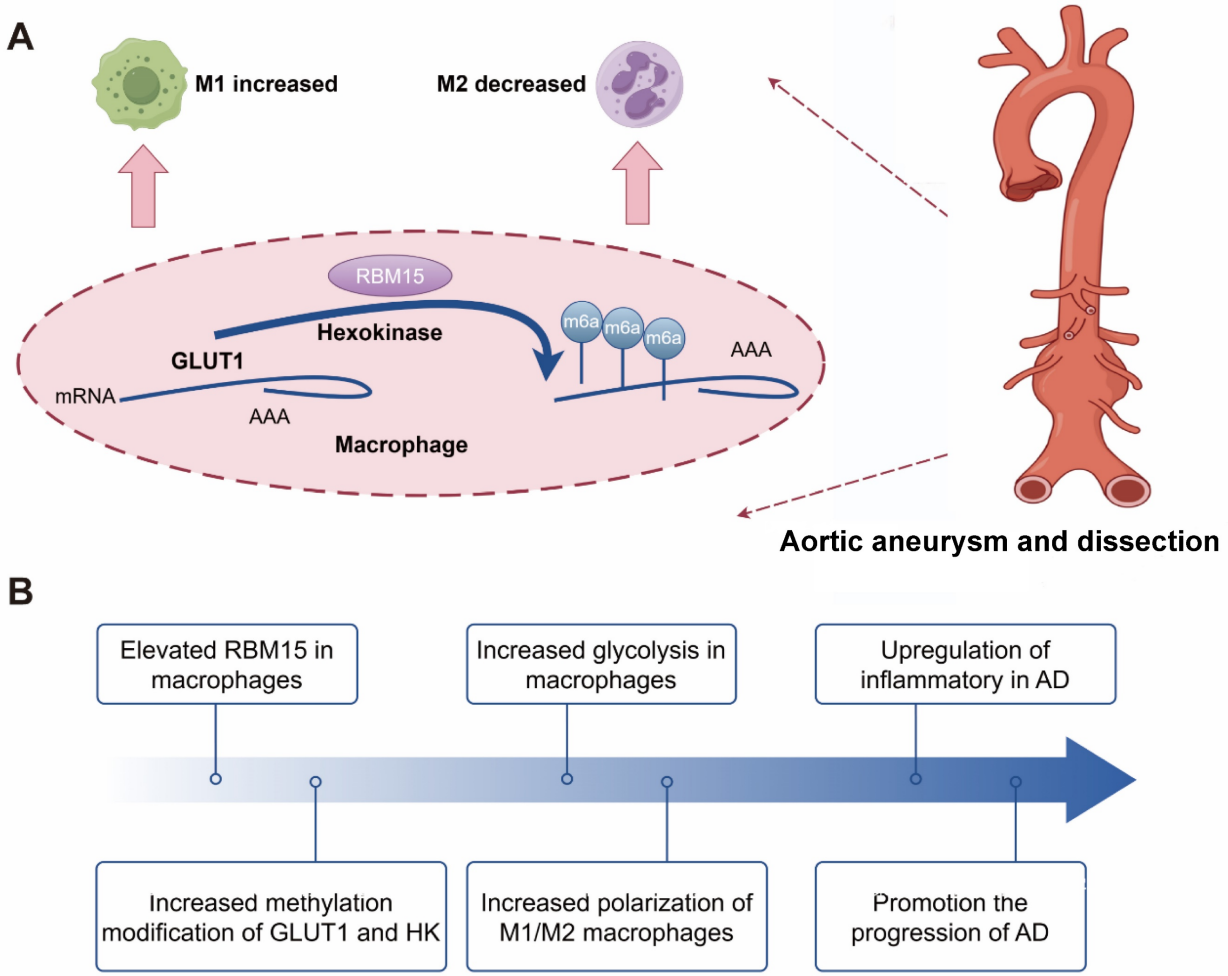
The following schematic representation depicts the hypothesized mechanism by which RBM15 activates glycolysis in M1-type macrophages, thereby promoting the development of AD. The m6A modification-related gene RBM15, which is highly expressed in AD samples, enhances glycolysis of macrophage, and affects polarization by promoting M1-type and decreasing M2-type markers. Knockdown of RBM15 alleviates AD by reducing M1-type macrophage markers and increasing M2-type macrophage markers.

Animal models play a crucial role in replicating the progression of AD and investigating its underlying mechanisms 51-53. In our study, we successfully established an AD model in SD rats and found that RBM15 knockdown mitigated the development of aortic aneurysms. Interestingly, we observed a reduction in the M1-type macrophage marker iNOS in the aorta, along with an increase in the M2-type macrophage marker CD206 following RBM15 knockdown. These findings highlight the important role of RBM15 in modulating macrophage polarization through glycolysis, which is intricately linked to the progression of AD (**Figure 9**). Our study provides insight into the complex interplay between AD pathology, m6A modifications, macrophage polarization, and metabolic reprogramming. RBM15 emerges as a significant biomarker in this intricate network of interactions, opening the door for further exploration and potential clinical applications in the field of AD therapeutics.

### Limitations

Our study has limitations. It is based on *in vitro* experiments and an animal model of AD, which may not entirely replicate the disease as seen in humans. RBM15's clinical significance in human AD patients has yet to be fully established and requires further investigation. Additional research involving human patients is necessary. Our study focuses on glycolytic metabolism, but the metabolic profile of macrophages in AD likely involves additional pathways, necessitating further exploration. Future studies should include a more comprehensive analysis of metabolic pathways in macrophages. Our study does not take into account the potential impact of other factors such as genetics, lifestyle, and environmental influences on the metabolic profile of macrophages in AD, which could have significant implications for future research.

## Conclusion

The research demonstrates that the dysregulation of the m6A-related gene RBM15 during the progression of AD has a significant impact on the expression of macrophage polarization markers. Moreover, it plays a crucial role in influencing macrophage metabolic reprogramming, particularly through glycolysis.

## Supplementary Material

Supplementary tables.

## References

[1] Tchana-Sato V, Sakalihasan N, Defraigne JO (2018). [Aortic dissection]. Rev Med Liege.

[2] Prêtre R, Von Segesser LK (1997). Aortic dissection. Lancet.

[3] Criado FJ (2011). Aortic dissection: a 250-year perspective. Tex Heart Inst J.

[4] Khan IA, Nair CK (2002). Clinical, diagnostic, and management perspectives of aortic dissection. Chest.

[5] Muzammil MA, Chaudhary N, Abbas SM, Ahmad O, Nasir A, Baig E (2024). Advancements in Serum Biomarkers for Early Diagnosis and Prognostic Assessment of Aortic Dissection. Crit Pathw Cardiol.

[6] Carbone A, Palladino R, Franzese M, Castaldo R, Ranieri B, Crisci G (2024). Health-related quality of life in patients with aortic dissection: An unmet need. Curr Probl Cardiol.

[7] Fanelli F, Cannavale A, O'Sullivan GJ, Gazzetti M, Cirelli C, Lucatelli P (2016). Endovascular Repair of Acute and Chronic Aortic Type B Dissections: Main Factors Affecting Aortic Remodeling and Clinical Outcome. JACC Cardiovasc Interv.

[8] Zou LW, Liu YF, Liu H, Chen B, Jiang JH, Shi Y (2024). [Surgical strategies and efficacy analysis for aortic dissection complicating intractable mesenteric artery ischemia]. Zhonghua Wai Ke Za Zhi.

[9] Calanchini M, Bradley-Watson J, McMillan F, Myerson S, Fabbri A, Turner HE (2024). Risk assessment for aortic dissection in Turner syndrome: The role of the aortic growth rate. Clin Endocrinol (Oxf).

[10] Pollari F, Nardi P, Mikus E, Ferraro F, Gemelli M, Franzese I (2024). Comparison of 4 mortality scores for surgical repair of type A aortic dissection: a multicentre external validation. Eur J Cardiothorac Surg.

[11] Sendinc E, Shi Y (2023). RNA m6A methylation across the transcriptome. Mol Cell.

[12] Karthiya R, Khandelia P (2020). m6A RNA Methylation: Ramifications for Gene Expression and Human Health. Mol Biotechnol.

[13] Qin Y, Li L, Luo E, Hou J, Yan G, Wang D (2020). Role of m6A RNA methylation in cardiovascular disease (Review). Int J Mol Med.

[14] Guo R, Dai J, Xu H, Zang S, Zhang L, Ma N (2022). The diagnostic significance of integrating m6A modification and immune microenvironment features based on bioinformatic investigation in aortic dissection. Front Cardiovasc Med.

[15] Yin F, Zhang H, Guo P, Wu Y, Zhao X, Li F (2022). Comprehensive Analysis of Key m6A Modification Related Genes and Immune Infiltrates in Human Aortic Dissection. Front Cardiovasc Med.

[16] Liao M, Zou S, Wu J, Bai J, Liu Y, Zhi K (2024). METTL3-mediated m6A modification of NORAD inhibits the ferroptosis of vascular smooth muscle cells to attenuate the aortic dissection progression in an YTHDF2-dependent manner. Mol Cell Biochem.

[17] de-Brito N M, Duncan-Moretti J (2020). Aerobic glycolysis is a metabolic requirement to maintain the M2-like polarization of tumor-associated macrophages. Biochim Biophys Acta Mol Cell Res.

[18] Zhu L, Zhao Q, Yang T, Ding W, Zhao Y (2015). Cellular metabolism and macrophage functional polarization. Int Rev Immunol.

[19] Kelly B, O'Neill LA (2015). Metabolic reprogramming in macrophages and dendritic cells in innate immunity. Cell Res.

[20] Yoshida GJ (2015). Metabolic reprogramming: the emerging concept and associated therapeutic strategies. J Exp Clin Cancer Res.

[21] Zhou D, Huang C, Lin Z, Zhan S, Kong L, Fang C (2014). Macrophage polarization and function with emphasis on the evolving roles of coordinated regulation of cellular signaling pathways. Cell Signal.

[22] Liu K, Zhao E, Ilyas G, Lalazar G, Lin Y, Haseeb M (2015). Impaired macrophage autophagy increases the immune response in obese mice by promoting proinflammatory macrophage polarization. Autophagy.

[23] Lee WJ, Tateya S, Cheng AM, Rizzo-DeLeon N, Wang NF, Handa P (2015). M2 Macrophage Polarization Mediates Anti-inflammatory Effects of Endothelial Nitric Oxide Signaling. Diabetes.

[24] Xie M, Yu Y, Kang R, Zhu S, Yang L, Zeng L (2016). PKM2-dependent glycolysis promotes NLRP3 and AIM2 inflammasome activation. Nature communications.

[25] Liu C, Liu C, Fu R (2022). Research progress on the role of PKM2 in the immune response. Front Immunol.

[26] Zhang B, Chen H, Ouyang J, Xie Y, Chen L, Tan Q (2020). SQSTM1-dependent autophagic degradation of PKM2 inhibits the production of mature IL1B/IL-1β and contributes to LIPUS-mediated anti-inflammatory effect. Autophagy.

[27] Van den Bossche J, O'Neill LA, Menon D (2017). Macrophage Immunometabolism: Where Are We (Going)?. Trends Immunol.

[28] Artyomov MN, Sergushichev A, Schilling JD (2016). Integrating immunometabolism and macrophage diversity. Semin Immunol.

[29] Liu Z, Le Y, Chen H, Zhu J, Lu D (2021). Role of PKM2-Mediated Immunometabolic Reprogramming on Development of Cytokine Storm. Front Immunol.

[30] Orecchioni M, Ghosheh Y, Pramod AB, Ley K (2019). Macrophage Polarization: Different Gene Signatures in M1(LPS+) vs. Classically and M2(LPS-) vs. Alternatively Activated Macrophages. Front Immunol.

[31] Juhas U, Ryba-Stanisławowska M, Szargiej P, Myśliwska J (2015). Different pathways of macrophage activation and polarization. Postepy Hig Med Dosw (Online).

[32] Mills CD (2012). M1 and M2 Macrophages: Oracles of Health and Disease. Crit Rev Immunol.

[33] Rath M, Müller I, Kropf P, Closs EI, Munder M (2014). Metabolism via Arginase or Nitric Oxide Synthase: Two Competing Arginine Pathways in Macrophages. Front Immunol.

[34] Pérez S, Rius-Pérez S (2022). Macrophage Polarization and Reprogramming in Acute Inflammation: A Redox Perspective. Antioxidants (Basel).

[35] Zhang Y, Zhang H, Zhao S, Qi Z, He Y, Zhang X (2024). S-Nitrosylation of Septin2 Exacerbates Aortic Aneurysm and Dissection by Coupling the TIAM1-RAC1 Axis in Macrophages. Circulation.

[36] Usman A, Massad MG (2014). The role of the macrophage in the development of aortic dissection. Cardiology.

[37] Luo F, Zhou XL, Li JJ, Hui RT (2009). Inflammatory response is associated with aortic dissection. Ageing Res Rev.

[38] Wang X, Zhang H, Cao L, He Y, Ma A, Guo W (2020). The Role of Macrophages in Aortic Dissection. Front Physiol.

[39] Zhou X, Chen Z, Zhou J, Liu Y, Fan R, Sun T (2021). Transcriptome and N6-Methyladenosine RNA Methylome Analyses in Aortic Dissection and Normal Human Aorta. Front Cardiovasc Med.

[40] Hu M, Yang Y, Ji Z, Luo J (2016). RBM15 Functions in Blood Diseases. Curr Cancer Drug Targets.

[41] Wang X, Tian L, Li Y, Wang J, Yan B, Yang L (2021). RBM15 facilitates laryngeal squamous cell carcinoma progression by regulating TMBIM6 stability through IGF2BP3 dependent. J Exp Clin Cancer Res.

[42] Yang F, Liu Y, Xiao J, Li B, Chen Y, Hu A (2023). Circ-CTNNB1 drives aerobic glycolysis and osteosarcoma progression via m6A modification through interacting with RBM15. Cell Prolif.

[43] Yuan J, Guan W, Li X, Wang F, Liu H, Xu G (2023). RBM15-mediating MDR1 mRNA m(6)A methylation regulated by the TGF-β signaling pathway in paclitaxel-resistant ovarian cancer. Int J Oncol.

[44] Feng J, Li Y, He F, Zhang F (2023). RBM15 silencing promotes ferroptosis by regulating the TGF-β/Smad2 pathway in lung cancer. Environ Toxicol.

[45] Yunna C, Mengru H, Lei W, Weidong C (2020). Macrophage M1/M2 polarization. Eur J Pharmacol.

[46] Yadav S, Dwivedi A, Tripathi A (2022). Biology of macrophage fate decision: Implication in inflammatory disorders. Cell Biol Int.

[47] Li X, Liu D, Zhao L, Wang L, Li Y, Cho K (2020). Targeted depletion of monocyte/macrophage suppresses aortic dissection with the spatial regulation of MMP-9 in the aorta. Life Sci.

[48] Gerloff D, Lützkendorf J, Moritz RKC, Wersig T, Mäder K, Müller LP (2020). Melanoma-Derived Exosomal miR-125b-5p Educates Tumor Associated Macrophages (TAMs) by Targeting Lysosomal Acid Lipase A (LIPA). Cancers (Basel).

[49] Viola A, Munari F, Sánchez-Rodríguez R, Scolaro T, Castegna A (2019). The Metabolic Signature of Macrophage Responses. Front Immunol.

[50] Sutula TP, Fountain NB (2023). 2DG and glycolysis as therapeutic targets for status epilepticus. Epilepsy Behav.

[51] Yang K, Ren J, Li X, Wang Z, Xue L, Cui S (2020). Prevention of aortic dissection and aneurysm via an ALDH2-mediated switch in vascular smooth muscle cell phenotype. Eur Heart J.

[52] Zheng HQ, Rong JB, Ye FM, Xu YC, Lu HS, Wang JA (2020). Induction of thoracic aortic dissection: a mini-review of β-aminopropionitrile-related mouse models. J Zhejiang Univ Sci B.

[53] Liu Y, Du J (2018). [Review on animal models of thoracic aortic aneurysm and dissection]. Zhonghua Xin Xue Guan Bing Za Zhi.

