# Supplementary material for: RBM15 activates glycolysis in M1-type macrophages to promote the progression of aortic aneurysm and dissection
Source: Int J Med Sci. 2024 Jul 22;21(10):1976–89. doi: 10.7150/ijms.97185 (PMC11302562; doi:10.7150/ijms.97185)
Supplement: Supplementary file 1 — Supplementary tables. [file ijmsv21p1976s1.pdf]

RBM15 activates glycolysis in M1-type macrophages to promote the progression of aortic aneurysm and dissection

Table S1. Primer sequences for RT-qPCR

METTL3	F:5′- TTGCATGGATTCTGAGGCCC-3′
	R:5′- GTCGGTCTGCACTGGAATCA-3′
METTL14	F:5′- CCAAAATCGCCTCCTCCCAA-3′
	R:5′- GTGGAAAGCCACCTCTGTGT-3′
METTL16	F:5′- AGAAAAAGCCCACCCCCAAA-3′
	R:5′- CAGAAGCCTCTTGGTCCTGG-3′
WTAP	F:5′- GTAATGGTAGCTCCTCCCGC-3′
	R:5′- CAAGCCATTCTGAACGTGGC-3′
KIAA1429	F:5′- ATGTTGTGCCACCACCAAGA-3′
	R:5′- ACCCACCACGGGAAGAAATC-3′
RBM15	F:5′- GTTATAGCTCCCCGAGCACC-3′
	R:5′- ACAAAGGCTACCCGCTCATC-3′
HNRNPC	F:5′- ACGTGTACCTCCTCCTCCTC-3′
	R:5′- TGCCCCTTCGTGAAGTGTTT-3′
FTO	F:5′- TTGCCCGAACATTACCTGCT-3′
	R:5′- TGTGAGGTCAAACGGCAGAG-3′
ALKBH5	F:5′- CCACGGATCCTGGAGATGGA-3′
	R:5′- TGACTTGCGCCAGTAGTTCT-3′
YTHDC1	F:5′- CCCCCTTACCCAGGAATGGA-3′
	R:5′- CTTGAGGAGGTGGAGCATGG-3′
HNRNPA2B1	F:5′- TGGAAATTATAACCAGCAACCTTCT-3′
	R:5′- CCATGTTCCTGCTACCACCA-3′
	F:5′- TTGGGGGCTCTCAATATGGC-3′

ZC3H13	R:5′- CAACGCCTTACAACATGGGC-3′
YTHDF1	F:5′- TGCACGATGCTGTTTTTGGG-3′
	R:5′- GCTGACGTCCCCAATCTTCA-3′
YTHDF2	F:5′- ATAGTTTGCCTCCAGCCACCACCA-3
	R:5′- GGACCGAAGCTTCTCCAACA-3′
YTHDF3	F:5′- GGGTCAGTGGTAAAGGCTCC-3′
	R:5′- GAGGAGCTACCCAGCGATTC-3′
IGF2BP1	F:5′- GGAAGTGAAGCTGGAGACCC-3′
	R:5′- TCCACCTTTGCCAATGACCC-3′
IGF2BP2	F:5′- AGAAGTCATCGTGCCTCGTG-3′
	R:5′- TGGCTAGCAAAGAAGTGCCC-3′
IGF2BP3	F:5′ - GCTCAGGGAAGAATTTATGGAAAAA-3′
	R:5′-AGCAAAGGATGGCACTCTGA-3′
CXCL9	F:5′- TGAGAAAGGGTCGCTGTTCC-3′
	R:5′-GGGCTTGGGGCAAATTGTTT-3′
CXCL10	F:5′-TGTACGCTGTACCTGCATCA-3′
	R:5′- GGACAAAATTGGCTTGCAGGA-3′
CXCL13	F:5′- TGTGTCCAAGAGAGCTCAGTC-3′
	R:5′- ATTCCCACGGGGCAAGATTT-3′
MRC1	F:5′-GGCTGCACTCTCATAGTGGG-3′
	R:5′-AAGCAGACCTTGGAATCGGG-3′
GLUT1	F:5′- GAGCATCATCTTCATCCCGGC-3′
	R:5′- GTTCTCCTCGTTGCGGTTGAT-3′
Hexokinase2	F:5′- AAGGACCTGGCTCCGAAATG-3′
	R:5′-CTATCGCTGTCCAGCCTCAC-3′
HIFα	F:5′- GCCAGACGATCATGCAGCTA -3′
	R:5′-TGCTCCATTCCATTCTGTTCAC -3′
GAPDH	F:5′- GGTCACCAGGGCTGCTTTTA-3′
	R:5′- TGAGGTCAATGAAGGGGTCA -3′

PFKFB3	F:5′- CAGCTGAAGAGCACCATCCA-3′	
	R:5′- GTCGATCTCATTGAGCGCCT-3′	

Table S2. Lv-RNA and sh-RNA sequences used in this study

Lv-RBM15	5′-CTGTAACGGAGAGTGATTTAA-3′
sh-RBM15	5′-ACTTACGGCTTTCTCAAATTT-3′
